# Investigation of Polyetherimide Melt-Extruded Fibers Modified by Carbon Nanoparticles

**DOI:** 10.3390/ma14237251

**Published:** 2021-11-27

**Authors:** Elena Ivan’kova, Gleb Vaganov, Andrey Didenko, Elena Popova, Vladimir Elokhovskiy, Alexander Bugrov, Valentin Svetlichnyi, Igor Kasatkin, Vladimir Yudin

**Affiliations:** 1 Institute of Macromolecular Compounds of Russian Academy of Sciences, V.O., Bol’shoy pr. 31, St. Petersburg 199004, Russia; glebvaganov@mail.ru (G.V.); vanilin72@yandex.ru (A.D.); men682003@mail.ru (E.P.); vladimir.elokhovskiy@gmail.com (V.E.); anbugrov@etu.ru (A.B.); valsvet@hq.macro.ru (V.S.); yudin@hq.macro.ru (V.Y.); 2 Department of Physical Chemistry, Saint Petersburg Electrotechnical University (ETU “LETI”), ul. Professora Popova 5, St. Petersburg 197376, Russia; 3 Research Centre for X-ray Diffraction Studies, Saint Petersburg State University, Universitetskaya nab. 7/9, St. Petersburg 199034, Russia; igor.kasatkin@spbu.ru

**Keywords:** polyetherimide, fibers, melt-extrusion, structure, rheology, VGCF, SEM, WAXS, DSC, mechanical properties

## Abstract

The fibers based on thermoplastic partially crystalline polyetherimide R-BAPB modified by vapor grown carbon nanofibers (VGCF) were prepared by melt extrusion, exposed to orientational drawing, and crystallized. All of the samples were examined by scanning electron microscopy, X-ray scattering, and differential scanning calorimetry to study how the carbon nanofiller influences on the internal structure and crystallization behavior of the obtained R-BAPB fibers. The mechanical properties of the composite R-BAPB fibers were also determined. It was found that VGCF nanoparticles introduced into R-BAPB polyimide can act as a nucleating agent that leads, in turn, to significant changes in the composite fibers morphology as well as thermal and mechanical characteristics. VGCF are able to improve an orientation degree of the R-BAPB macromolecules along the fiber direction, accelerate crystallization rate of the polymer, and enhance the fiber stability during crystallization process.

## 1. Introduction

Polyimides (PI) are a class of polymers with imide rings in the main chain, which can be aliphatic, aromatic, or semi-aromatic in composition [1,2]. Aromatic polyimides are distinguished by increased thermal stability, low coefficient of linear thermal expansion, and high mechanical and dielectric properties, as well as good radiation and chemical resistance [3,4]. On the basis of PI, films, coatings, varnishes, composites, binders, and foams are obtained [5]. In addition, they can be spun into fibers with exceptional technological and operational properties [6]. The production of continuous polyimide fibers with a large surface area to volume ratio and the possibility of their orientation during spinning have made it possible to obtain promising, new nonwovens used as fire-resistant fabrics, heat shields, high-temperature exhaust filters, air purification agents, scaffolds for tissue engineering, and various prepregs [7,8,9].

Typically, dry or wet spinning, as well as melt extrusion, are used to produce PI-based fibers [10]. Dry spinning involves passing a solution of polyimide or its prepolymer through a spinneret and blowing hot air on the forming fiber to remove the solvent, followed by heat treatment [11]. In wet spinning, the solution is passed through a coagulation bath with a precipitant in which the solvent is removed and the prepolymer is precipitated in the form of a fiber [12]. Finally, melt-spun fibers are produced by extrusion and drawing the finished PI above its melting point [13]. Extrusion of thermoplastic polyimides from a melt is more preferable, since it excludes the use of solutions, coagulation bath, and additional processing in order to remove volatiles and achieve thermal imidization of fibers [14]. However, polyimide fibers were rarely obtained by this method due to the high glass transition temperature due to the high rigidity of polymer chains and strong intermolecular interactions between them [15]. In recent years, by introducing flexible ether units into polymer chains, a number of polyetherimides have been synthesized, which made it possible to process them by melt extrusion [16].

Researchers from Teijin Chemicals Ltd. tried to produce polyesterimide fibers by melt-spinning process at 345–475 °C, but filament mechanical properties were unsatisfactory [10]. In 1983, polyimide fibers with a tensile strength of 1.6 GPa and an elastic modulus of 48 GPa, made by melt technology at 300–400 °C, were patented [17]. One study, [18], reported on melt-spun polyesterimide fibers with an elongation at break of 570% and good water resistance. Fay C.C. et al. first described melt spinning of crystallizable polyimide fibers LaRCTM-IA based on 3,4′-oxydianiline and 4,4′-hydroxyphthalic anhydride terminated at the end groups with phthalic anhydride [19]. At a draw ratio of 3.9, the tensile strength (σ) of LaRCTM-IA fibers was 159 MPa, the elastic modulus (E) reached 3.21 GPa, and the elongation at break (ε) was 103%. The amorphous polyimide fibers ULTEM obtained as a reference in the same work based on bisphenol A dianhydride and m-phenylenediamine at average draw ratios from 4.5 to 10.3 gave σ values from 138 to 207 MPa, E from 2.7 to 3.2 GPa, and ε from 47 to 111%.

High mechanical characteristics (σ = 135.3 ± 12.5 MPa; E = 2.7 ± 1.1 GPa; ε = 127 ± 8%) were also recorded for R-BAPB semi-crystalline polyimide fibers based on 1,3-bis-(3,3′,4,4′-dicarboxyphenoxy) benzene and 4,4′-bis (4″-aminophenoxy) biphenyl at their 4-fold drawing in the amorphous state [20,21].

The possibility of increasing the strength, as well as a number of other characteristics of polyimides, by introducing into them carbon nanoparticles, such as nanotubes and nanofibers, is of particular interest. A distinctive feature of these nanoparticles is a high specific surface area of the nanoparticles, the possibility of varying their axial ratio, and chemical surface modification. It is known that the incorporation of the carbon nanoparticles into the PI matrix can improve the mechanical and thermophysical properties of moldings, carbon reinforced plastic, and films [22,23]. A melt-mixing method was used to fabricate PI-based nanocomposites with 1 wt.% to 3 wt.% carbon nanofibers (CNFs) [24]. The composites with 1 wt.% CNFs showed flexural strength and toughness increased by more than 50% and 550%, respectively. The introduction of the carbon nanoparticles into the PI matrices leads not only to a change in the mechanical properties, but also to a change in the structure of the PI composite. A carbon nanoparticle can lead to the initialization and acceleration of the crystal growth of PI [25]. In addition, a strong orientation of the crystallizable PI macromolecules near the surface of the carbon nanoparticles was shown in the literature by the simulation method, which, in turn, leads to an improvement in the mechanical properties of the PI nanocomposites [26,27].

It is extremely important to note that most of the previously published works on PI nanocomposites are focused on block samples obtained from PI melt. Only in [28], oriented fibers were obtained from polyimide (ULTEM) melt, modified with single-walled carbon nanotubes (SWCNT) in an amount from 0.1 to 1 wt.%. It is shown that the introduction of 1 wt.% SWCNT leads to an insignificant increase in the mechanical properties of the PI fibers (by ≈ 10% of strength and elastic modulus) giving, as a result, an elastic modulus of 3.2 GPa, a tensile strength of 105 MPa, and a deformation at break of 20%. Probably, such a slight increase in the mechanical properties of the PI-based fibers is associated with the complexity of the uniform distribution and destruction of SWCNT aggregates in the PI melt to a single nanoscale state. However, the mechanical properties achieved in melt-extruded PI fibers are far from those of wet-spun fibers. For example, PI composite fibers containing 1 wt.% carbon nanotubes and having a strength of 1.5 GPa and an elastic modulus of 58.5 GPa were prepared in by the traditional method of wet spinning from polyamidoacid followed by thermal imidization [29]. To realize the maximum possible mechanical characteristics of the fiber, in addition to its modification with carbon nanoparticles, it was necessary to achieve maximum orientation and straightening of the macromolecules along the fibers, for example, due to the process of thermal stretching. Using R-BAPB semi-crystalline fibers as an example, it was shown in [21] that an increase in the SWCNT concentration from 0.05 to 0.2 wt.% results in an increase in the degree of their crystallinity from 42.8 to 45.1%. In this case, the highest values of the strain-strength properties (σ = 142.7 ± 5.9 MPa; E = 5.2 ± 0.1 GPa; ε = 6.1 ± 0.3%) of the R-BAPB/SWCNT composite fibers were observed at a filling degree of 0.1 wt.%. Although an increase in the mechanical properties of the PI fibers with the incorporation of SWCNT is recorded in the literature, they are far from the maximum possible values predicted by the reinforcing ability of one-dimensional carbon inclusions.

When obtaining PI fibers from a melt, the most interesting is the group of partially crystalline PI due to the presence of a crystalline phase, that makes it possible to increase the thermal stability, wear resistance, and resistance to solvents of the obtained PI material. One of the promising carbon fillers for such PI fibers could be carbon nanofibers. Carbon nanofibers obtained by vapor deposition (VGCF) have a high axial ratio, are easily dispersed, and more accessible than carbon nanotubes. This was the main reason for the attempt to obtain composite fibers based on R-BAPB polyimide filled with VGCF nanoparticles by melt extrusion and to track changes in their structure-property relationships.

In this regard, the purpose of this work was a detailed study of changes in the structure and a number of different properties (mechanical and thermal) of the fibers based on crystallizing polyimide R-BAPB modified by vapor grown carbon nanoparticles (VGCF), followed by orientational thermal drawing, as well as additional heat treatment leading to crystallization of the polymer matrix.

## 2. Materials and Methods

The heat-resistant thermoplastic R-BAPB polyimide (PI) was synthesized in the powder form on the basis of dianhydride R (1,3-bis-(3′,4-dicarboxyphenoxy) benzene), T_m_~164 °C, (OOO TechHimProm, Yaroslavl, Russia) and diamine BAPB (4,4′-bis(4″-aminophenoxy) biphenyl), T_m_~198–199 °C, of VWR International manufacturer. Phthalic anhydride was chosen as a limiting agent of chain growth for polycondensation reaction, T_m_~131–134 °C (Sigma-Aldrich Co. LLC, St. Louis, MO, USA). Acetic anhydride, benzene, and triethylamine were supplied by Sigma-Aldrich Co. LLC. Vapor grown carbon nanofibers (VGCF) with a diameter of about 150 nm and a length of about 10–20 μm (Showa Denko, Tokyo, Japan) were added to polyimide R-BAPB for its modification.

To synthetize polyimide R-BAPB, the method of chemical imidization was used in the present work. At the first stage of the synthesis, the resorcinic anhydride R polycondensation reaction with diamine BAPB into polyamic acid (PAA) in a solution of an amide solvent (N-methylpyrrolidone) was carried out. The process of chain growth was terminated by adding phthalic anhydride into the reaction mixture in order to chemically deactivate the terminal groups of the macromolecules. The ratio of dianhydride to diamine was maintained for molecular weight control. The molar ratio of the monomers (R to BAPB) was 0.95:1. A VGCF suspension in N-methylpyrrolidone (MP) was admixed to the obtained PAA. Next, the chemical PAA cyclization was fulfilled according to the protocol described elsewhere [30].

**Figure 1:**
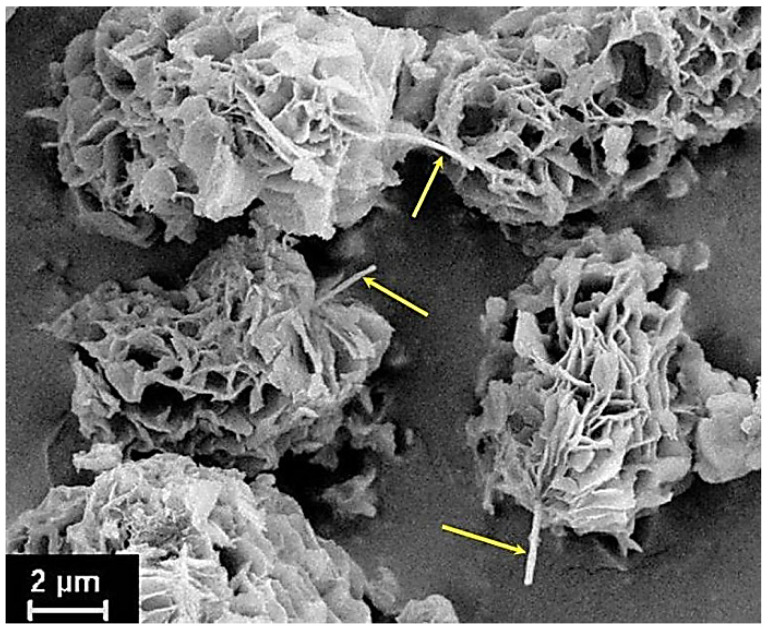
Polyimide R-BAPB nascent powder particles synthesized with added VGCF nanofibers (VGCFs are shown by yellow arrows).

In order to obtain nanocomposite fibers, the calculated amount of VGCF nanofibers was loaded into the resulting PAA solution. The VGCFs were dispersed in the PAA solution using an overhead stirrer for 12 h. Then, the resulting suspension of VGCF nanoparticles in a PAA solution was subjected to chemical imidization by analogy with pure PAA. The resulting powders were dried for 2 h at 220 °C under vacuum. The concentrations of VGCF were equal to 0.5, 1, and 2 wt.%. Figure 1 shows the scanning micrograph of the synthesized polyimide R-BAPB powder particles with VGCF nanofibers included inside them.

For melt extrusion of R-BAPB polyimide-based fibers, a DSM Xplore twin-screw microextruder (Xplore Instruments, Sittard, The Netherlands) equipped with a special fiber preparation unit (DSM Film Device Machine, Xplore Instruments, Sittard, The Netherlands) was used. The synthesized PI powder was loaded into a microextruder heated to 360 °C. The molten mass was mixed at a temperature of 360 °C for 5 min for air removing and melt homogenization at the screw rotation speed of 50 rpm. The fiber was extruded at 25 rpm through a round die with a diameter of 1 mm. After passing the spinneret, the fiber was cooled down by an air stream and wound onto the receiving coil at a constant speed. As a result, a monofilament fiber was formed based on the R-BAPB polyimide in amorphous state. These fibers were further drawn with the help of a stretching line with heating element at 230 °C, up to draw ratio DR = 4. To crystallize the R-BAPB polyimide fibers, an additional annealing was conducted at the temperature of 280 °C for 1 h.

The polymer melt viscosity was controlled by a rheometer Physica MCR301 (Anton Paar, Graz, Austria) with a cone-plane measuring unit CP25-2 (diameter 25 mm, angle 2°, the cone-plane gap was 0.05 mm). The test was performed in shear mode at 360 °C and shear rates varied from 1 to 0.01 s^−1^.

The VGCF nanofibers distribution in the R-BAPB polyimide matrix inside the composite fibers was reviewed by a scanning electron microscope SUPRA-55VP (Carl Zeiss, Oberkochen, Germany) in a secondary electron mode. The investigated fibers were cryo-cleaved at temperature of liquid nitrogen. The longitudinal and cross-sections were fixed on the special microscope holders and sputtered by a thin layer of Pt.

Examination of the crystalline structure of the R-BAPB-based fibers was carried out by WAXS with the help of the Diffractometer D8 Discover (Bruker, Karlsruhe, Germany) with point focus, 0.5 mm spot size and parallel beam filtered CuKα radiation, as well as an Imagine Plate area detector (Anton Paar, Graz, Austria). Azimuthal intensity distribution profiles were recorded for the 002 graphite diffraction peaks for the R-BAPB polyimide nanocomposite fibers before and after drawing.

The thermal properties of the prepared R-BAPB-based fibers were investigated by the differential scanning calorimetry (DSC) method with the help of DSC 204 F1 device (NETZSCH, Selb, Germany). The experiments were made in the temperature range 30–300 °C at the heating rate of 10 °C/min, in inert atmosphere of argon. The weight of the samples was 4 mg. For calculation of the degree of crystallinity of the obtained fiber samples, the value of melting enthalpy (ΔH^0^m) was used, which was previously determined for R-BAPB with the crystallinity degree of 100% as 90 J/g [31].

Mechanical tests in a tension mode were performed with the help of a universal tensile testing machine INSTRON 5943 (Instron, High Wycombe , UK), according to the ISO 527 Standard at the test speed of 10 mm/min. A total of 10 samples, with a base length of 30 mm, were examined for each fiber type. The values of tensile strength, Young’s modulus and deformation at break were obtained from the recorded stress-strain curves. Strain range for linear fit for Young’s modulus calculation was 0.025–0.25%. The measurement error did not exceed 15%.

## 3. Results and Discussion

### 3.1. Rheological Studies of PI Melts Based on R-BAPB and Their Mixtures with Nanoparticles

The most important condition for the successful molding process is an optimal level of melts viscosity. At very high viscosity, disruption of the flow continuity with the appearance of breaks in the melt jet is very possible. In this regard, it was necessary to make a scientifically sound selection of the optimal conditions for obtaining fibers based on R-BAPB partially crystalline polyimide by the melt method.

**Figure 2:**
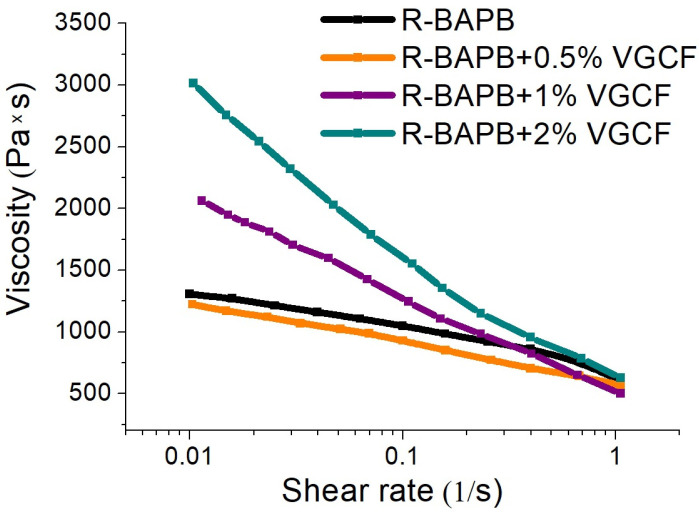
Dependence of viscosity on shear rate for R-BAPB modified with VGCF carbon nanoparticles.

Figure 2 presents the dependence of the melt viscosity on the applied shear rate for the pure R-BAPB polyimide as well as for its compositions with VGCF. All investigated compositions exhibit a non-Newtonian character during flow. The introduction of carbon nanoparticles into a polymer matrix based on R-BAPB leads to an increase in viscosity at a low shear rate, that is clearly observed at a concentration higher than 1 wt.% for the melt mix of R-BAPB with VGCF. This viscosity enhancement can be ascribed to the formation of a structural network of the nanofibers loaded in the polyimide matrix [32,33]. Increasing shear rate leads to destroying the bonds in such VGCF network that results in a reduction in the viscosity of the compositions. Furthermore, a structural network is probably formed due to the interaction directly between VGCF as well as through the matrix layers. Such essential rise of the viscosity at low shear rates can be explained by the following fact: by virtue of the high degree of the nanofibers anisometry (the ratio of VGCFs length to their diameter is ~30–100) and a rather large specific surface (~13 m^2^/g), there is a strong interaction of the polymer macromolecules with the nanofiber’s surface. Finally, this results in the formation of a stronger structural VGCF network inside the R-BAPB matrix and, as a consequence, a sufficiently high shear effort is necessary for its disruption. Therefore, it can be concluded that the VGCF particles are well dispersed in the polyimide matrix.

It should be noted that the fiber spinning occurred at the rates higher than 1 s^−1^. At these rates, the melt viscosity of the carbon nanoparticle modified polyimide became acceptable for spinning of all kinds of the R-BAPB-based fibers.

As mentioned earlier, the introduction of 1 wt.% VGCF or more into the R-BAPB polyimide leads to a significant increase in the viscosity of the polymer melt. However, this viscosity value still allowed the formation of the fibers. The higher content of the carbon nanoparticles was accounted for very high values of the melt viscosity, which made it impossible to obtain a high-quality defect-free fiber during the processing of the studied polyimide by the melt method. However, for further comparison of several properties, fibers based on R-BAPB with other concentrations of nanoparticles (0.5 wt.% and 2 wt.% VGCF) were also prepared.

### 3.2. Scanning Electron Microscopy

**Figure 3:**
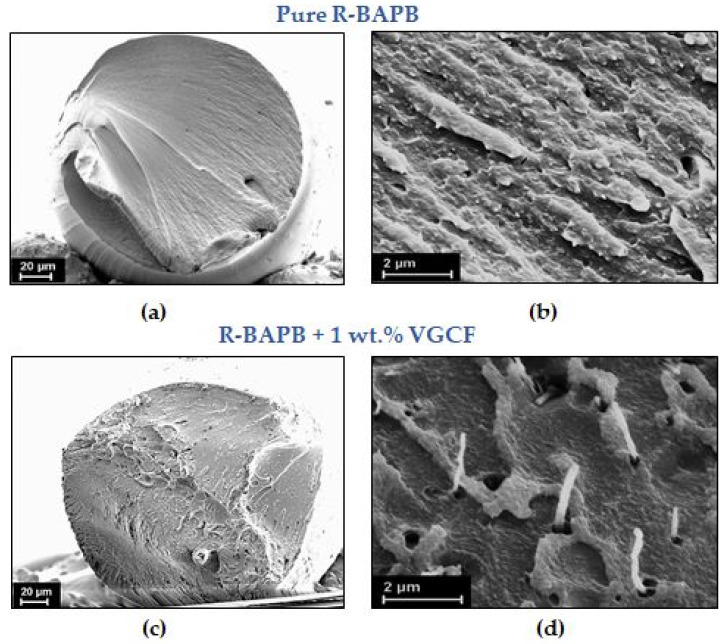
SEM images at different magnification of cryo-cleavages of the unoriented amorphous fibers (DR = 1) based on R-BAPB polyimide with the addition of 1 wt.% VGCF. (**a**,**b**) the pure R-BAPB fiber, (**c**,**d**) the composite R-BAPB fiber filled with 1 wt.% VGCF.

As noted above, it is also extremely important to control the dispersion of the nanofiller in the final product, that is, in the resulting composite fibers. In this regard, it is necessary to visualize how the VGCF nanoparticles are distributed in the volume of the composite R-BAPB fibers (Figure 3).

As shown Figure 3, the VGCF nanoparticles are distributed fairly evenly in the polymer R-BAPB matrix. A number of small aggregates consisting of several nanoparticles were found inside the fibers with 1 wt.% VGCF. When the composite fiber breaks, the nanoparticles can be pulled out of the polyimide matrix, which means insufficient adhesion between the polymer and nanoparticles. Consequently, the particle-polymer interfacial regions can be viewed as numerous defects within the composite material.

**Figure 4:**
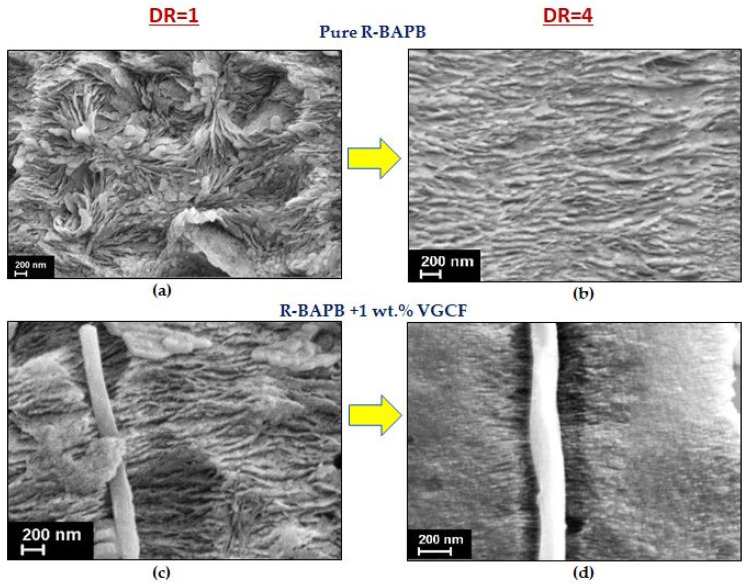
SEM images of the surface of crystallized fibers based on R-BAPB polyimide with the addition of 1 wt.% VGCF before (DR = 1) and after orientational drawing (DR = 4). The direction of fiber orientation was vertical. The pure R-BAPB fiber: (**a**) before orientation drawing DR = 1, (**b**) after drawing up to DR = 4; the composite R-BAPB fiber filled with 1 wt.% VGCF: (**c**) before orientation drawing DR = 1, (**d**) after drawing up to DR = 4.

In addition, the SEM method was also used to study the structure of the composite fibers based on polyimide R-BAPB, which underwent additional thermal annealing to transition from an amorphous to a partially crystalline state, both before (DR = 1) and after orientation drawing (DR = 4). These results are shown in Figure 4. To visualize the internal structure of crystallized fibers, they were subjected to selective chemical etching using a KMnO_4_ solution in H_3_PO_4_ acid [34].

It is well seen that before orientational drawing, the crystallized fiber made of pure polyimide R-BAPB consists of weakly oriented lamellae, while after passing through the stage of high-temperature orientational drawing, the partially crystalline lamellae are oriented perpendicular to the fiber axis (and, accordingly, normal to the drawing direction). VGCF nanoparticles are found to be oriented along the fiber axis during extrusion. It is worthy to note, that the introduction of the anisometric carbon nanofibers into the polymer R-BAPB matrix leads to the appearance of normally oriented polymer lamellas in the crystallized sample that has not yet been subjected to orientational drawing (DR = 1). This fact, presumably, may indicate that this nanofiller has a nucleative effect on the R-BAPB matrix.

### 3.3. Wide-Angle X-ray Scattering (WAXS)

**Figure 5:**
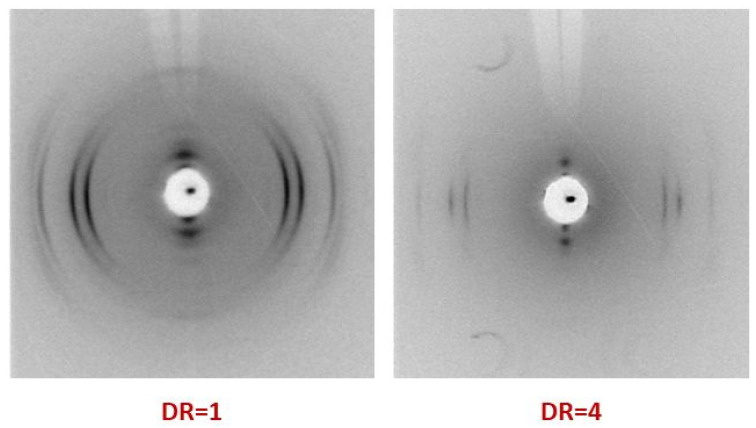
WAXS diffraction patterns of the crystallized R-BAPB fibers before and after orientational drawing (the fiber axis was vertical).

The study of the fine structure of the crystallized nanocomposite fibers based on R-BAPB polyimide before and after orientational drawing was carried out using the wide-angle X-ray scattering (WAXS) method. Figure 5 shows examples of X-ray photographs of the R-BAPB polymer fibers (in all cases, the fiber was placed vertically).

Based on the WAXS data presented in Figure 5, one can conclude that after additional thermal annealing, the fibers have a crystal structure typical of this polymer. Leaving the die of the extruder, the fiber already has a certain preferential orientation of macromolecules along its axis, which subsequently leads to the appearance of texture in the crystallized state. Therefore, in Figure 5 on the left side (DR = 1), instead of Debye rings, arches of the main crystal R-BAPB reflections are revealed. Moreover, after orientational drawing up to DR = 4, a noticeable narrowing of the arched reflections is observed, which indicates a significant improvement in the orientation of the polymer crystallites along the fiber axis.

To assess the degree of misorientation of the crystalline regions in the R-BAPB polymer matrix, azimuthal profiles of equatorial reflections were obtained. The half-width of this azimuthal reflex (Δφ°) will give us the desired degree of misorientation.

**Figure 6:**
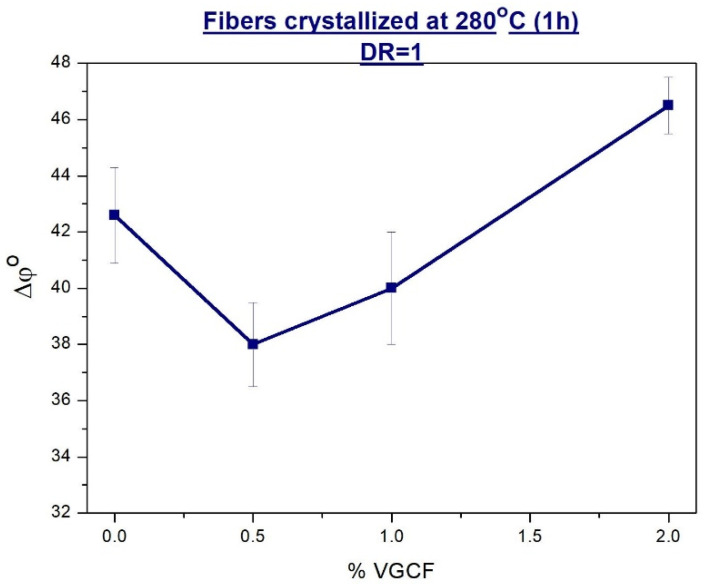
Dependences of the misorientation angle (along the fiber axis) of the crystallites on the content of the nanoparticles in the unoriented (DR = 1) composite fibers based on R-BAPB.

An effect of the loaded VGCF nanoparticles into the R-BAPB polymer matrix on the misorientation angle (Δφ°) along the axis of the unoriented (DR = 1) crystallized nanocomposite fiber is clearly demonstrated in Figure 6. It is well seen that a small amount of the nanofiller (0.5 wt.% VGCF) improves the alignment of the polyimide crystallites in the direction of extrusion. However, further increase in the nanofibers content gives an opposite result, leading to a rise of the misorientation degree. The reason of such structural changes at VGCF concentration >1 wt.% could be ascribed to the fact that the VGCF nanoparticles added in high amount cannot be effectively oriented being in the melt and passing through the die hole. Therefore, the polymer crystallites nucleated on them also are far from being perfectly oriented. Hopefully, the following orientational drawing at higher temperatures could help the polymer composition to better orientation of its components.

Nevertheless, on the basis of WAXS data similar to those presented in Figure 5, it is possible to calculate the transversal dimensions of the R-BAPB crystallites for all studied samples.

**Figure 7:**
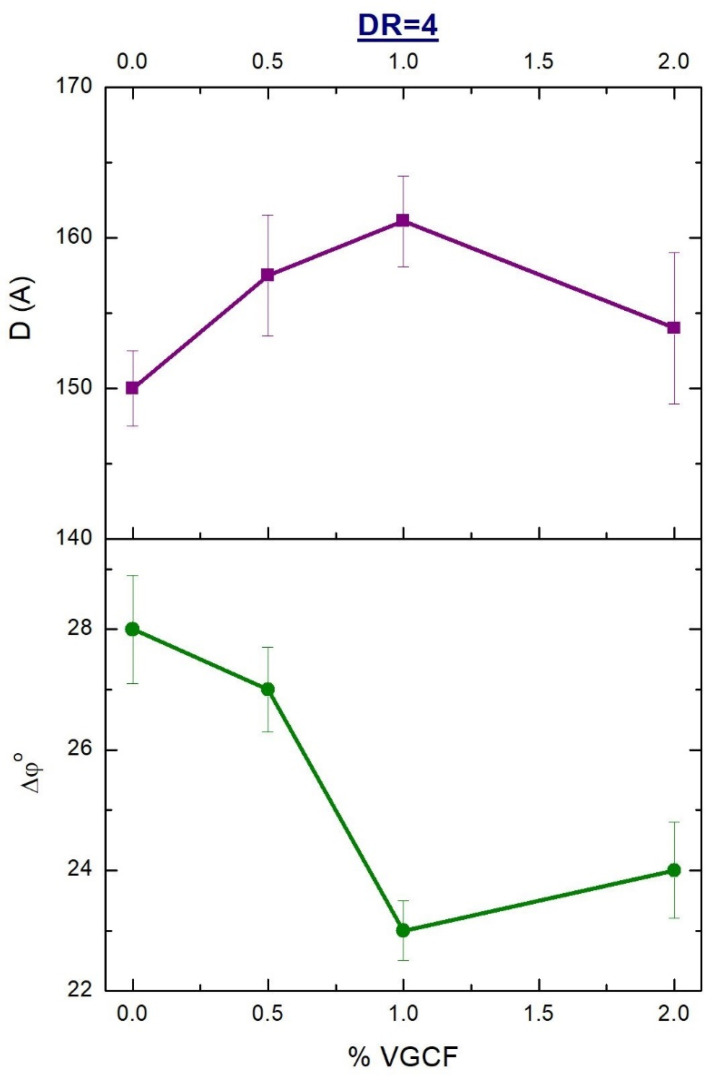
Dependences of the transverse size and misorientation angle (along the fiber axis) of the crystallites on the content of the nanoparticles in the ultimately oriented (DR = 4) composite fibers based on R-BAPB.

Figure 7 shows the concentration dependences of the transverse dimensions of the crystallites and the angle of the crystallites misorientation in the oriented (DR = 4) fibers based on polyimide R-BAPB filled with VGCF. With an increase in the VGCF concentration up to 1 wt.%, the half-width of this azimuthal reflex (Δφ°) decreases, indicating an improvement in the orientation of the crystalline regions (and, accordingly, of the polyimide macromolecules inside them) along the fiber axis. A further increase in the content of the nanoparticles leads to the opposite effect, namely, a slight increase in the misorientation angle Δφ°. The observed effects can be explained by the fact that, at a low content of the filler, its nanoparticles can be quite easily oriented along the extrusion direction and the following orientational drawing, being in the field of mechanical forces. In its turn, playing the role of the nucleants for polyimide R-BAPB, the nanofibers contribute to an improvement in the orientation of the polymer crystallites. When the filler content exceeds certain levels (>1 wt.% VGCF), nanoparticles begin to interfere with each other’s orientation along the fiber axis, which leads to a deterioration in the orientation of both the nanoparticles and the R-BAPB crystallites nucleated on their surfaces.

As noted above, the transverse sizes of the polymer crystallites in the studied samples were also calculated (see Figure 7). Judging by the received calculations, the dependence of the crystallite size in the polyimide matrix on the nanoparticle concentration is nonlinear and decreases after exceeding the optimal levels (1 wt.% VGCF). This, in turn, can further affect the mechanical properties of the resulting fibers and their operation at elevated temperatures. Thus, a preliminary conclusion can be drawn about the optimal concentrations of the nanoparticles introduced into the polyimide matrix, namely 1 wt.% VGCF.

**Figure 8:**
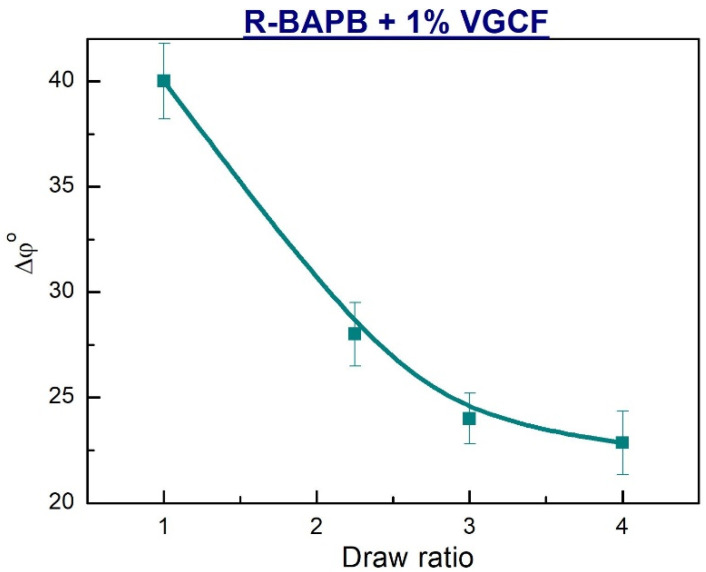
Dependences of the angle of misorientation (along the fiber axis) of the polymer crystallites on the draw ratio of the composite fibers based on R-BAPB loaded with 1 wt.% VGCF.

Taking into account the data mentioned above and the conclusions drawn on their basis, the dependences of the misorientation angle Δφ° on the draw ratio of the nanocomposite fibers with the content of 1 wt.% VGCF were obtained (Figure 8). An analysis of the plotted dependences showed that an increase in the degree of drawing of the samples up to DR = 4 makes a significant contribution to the improvement of the orientation of the R-BAPB crystallites. This fact is of particular importance, since it means that in the process of orientational drawing there is an effective alignment of the macromolecules in the direction of the fiber axis, rather than their slipping along to each other.

### 3.4. Differential Scanning Calorimetry (DSC)

**Figure 9:**
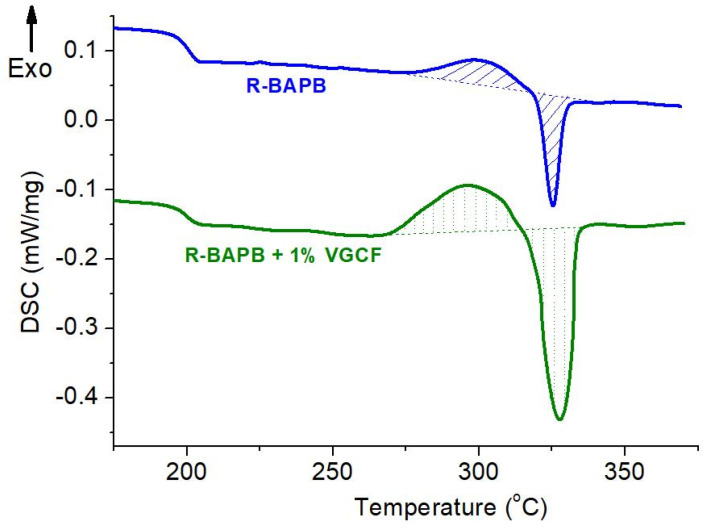
DSC thermograms of the unoriented (DR = 1) amorphous fibers of polyimide R-BAPB with the VGCF nanofillers.

The method of differential scanning calorimetry was used to study the thermophysical properties of the melt-extruded fibers based on polyimide R-BAPB and modified with the VGCF nanoparticles, as well as to obtain additional information necessary for choosing the conditions for orientational drawing and subsequent thermal annealing of the fiber samples. The obtained results are reflected in Figure 9.

According to the data shown in Figure 9, the VGCF carbon nanoparticles introduced into the R-BAPB fibers have a nucleating ability. This effect manifests itself in the form of a decrease in the temperature of the onset of crystallization (in comparison with the fiber made of pure polyimide R-BAPB) and an increase in the degree of crystallinity, i.e., areas under the peaks of crystallization and melting. It was also found from isotherms obtained at 280 °C for the pure R-BAPB and composite fibers that the introduction of the carbon nanofibers significantly accelerates the crystallization of the unoriented (DR = 1) fibers based on R-BAPB polyimide: loading of only 1 wt.% VGCF reduces the crystallization time by a factor of 5. Therefore, it can be said with confidence that adding of the carbon nanofillers leads to an earlier onset and acceleration of the crystallization process of the polyimide matrix.

**Figure 10:**
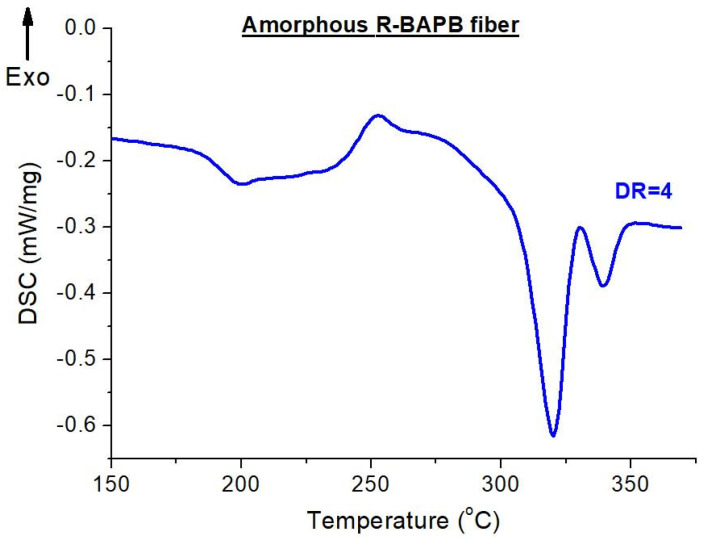
DSC thermogram of the amorphous polyimide R-BAPB fiber after orientation drawing (DR = 4).

High-temperature orientational drawing of the composite fibers based on R-BAPB (see Figure 10) leads to the appearance of strongly diffuse crystallization and melting peaks (we will not give a detailed description of the processes resulting in the presence of double melting peaks here—this will be published in the next paper). Since the temperature range, in which the polymer matrix crystallizes, is very wide, it makes no sense to present the crystallization temperature. However, the data obtained by the DSC method provide us with extremely important information about the temperatures, at which the next stage of the sample preparation should be carried out, namely, thermal annealing in order to crystallize the polymer matrix. In this work, a careful selection of conditions (temperatures and times) was carried out for additional heat treatment of the investigated composite fibers in order to achieve the maximum possible degrees of crystallinity. In addition, while the oriented (DR = 4) fibers were isothermally annealed, it was found that the fibers made of pure R-BAPB had strong shrinkage during crystallization process and most of them ruptured without having time to crystallize. Due to this observation, we had to reduce the starting temperature point of crystallization to 230 °C. In the course of the work, it was found that the introduction of the nanoparticles allows the fibers to retain their shape during annealing and to crystallize these composite fibers as much as possible (the degree of crystallinity reached 35%), which should further lead to an increase in the heat resistance of these fibers. Ultimately, the following scheme was chosen: Stage 1—quick heating from T_room_ up to 230 °C; Stage 2—slow heating up to 280 °C; and Stage 3—isotherm at 280 °C for 1 h.

**Figure 11:**
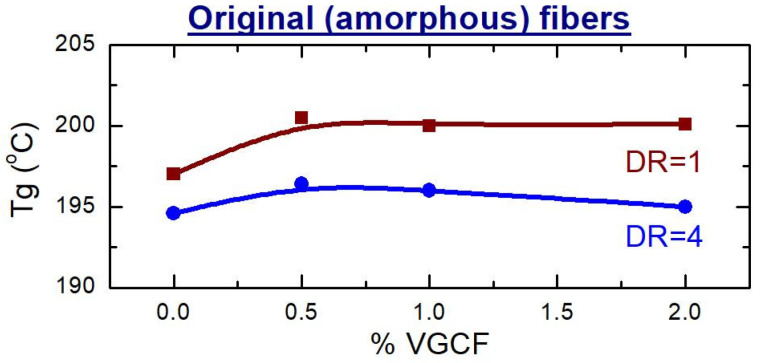
Dependences of the glass transition of the amorphous composite fibers before (DR = 1) and after (DR = 4) orientational drawing on the content of the nanofibers.

**Figure 12:**
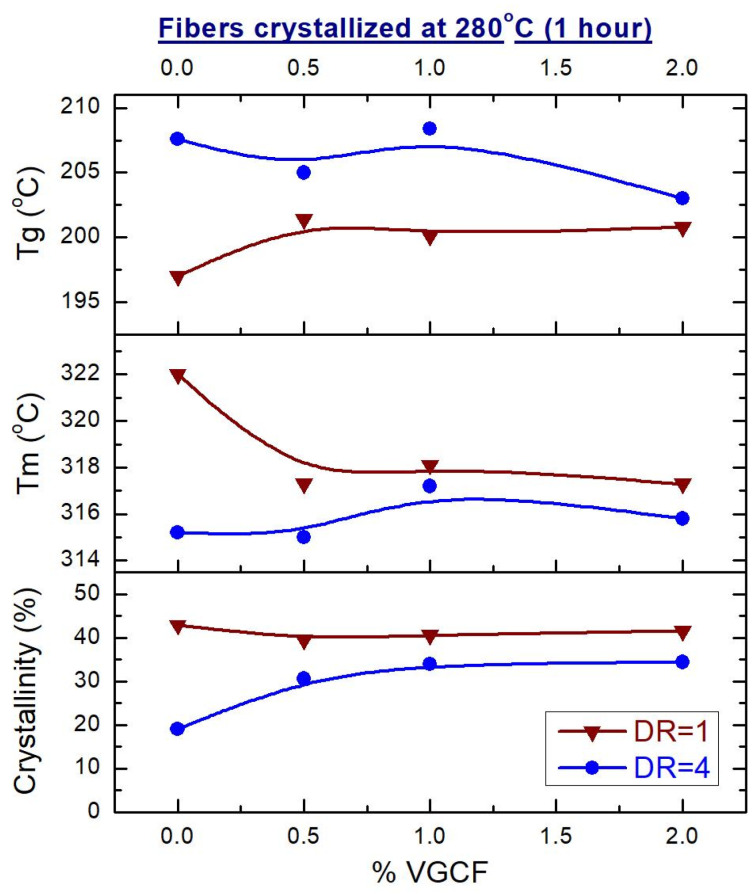
Concentration dependences of the glass transition and melting temperatures, as well as the degree of crystallinity of the crystallized composite fibers before (DR = 1) and after (DR = 4) orientational drawing on the VGCF content.

From the recorded DSC curves, the values of glass transition temperature (T_g_), melting point (T_m_), and the degree of crystallinity were obtained and plotted in Figure 11 and Figure 12.

Analyzing the obtained DSC data, it was evidently revealed that the glass transition temperature (Tg) of the unoriented amorphous samples (DR = 1) is higher than the Tg of the fibers after orientation (Figure 11). Most likely, this is due to the fact that the oriented state of the polyimide macromolecules is nonequilibrium one and, as a consequence, when the temperature rises, such macromolecules tend to return to their original state as soon as possible. As for the unoriented fibers, the VGCF nanoparticles are found to be able to restrict the mobility of the polyimide macromolecules and to act as macroscopic cross-links, inhibiting the mobility of the macromolecules. As results, it leads to slight increase in Tg of the composite fibers comparing to the pure R-BAPB fiber (Figure 11 and Figure 12). Similar effect was already observed elsewhere [35].

The melting temperature (T_m_) of the crystallized composite fibers, which had not yet been subjected to orientation drawing (DR = 1), drops when the VGCF nanoparticles are loaded in the polyimide matrix (Figure 12). It is known [36] that the melting of polymer crystallites begins from the lateral surfaces of the defect (end of the chain, folds, etc.) and occurs by separating sections of the macromolecules in the direction perpendicular to the crystal axis. But the greater the number of the introduced VGCF particles and the larger their surface are, the more transcrystallites adjoin them with their lateral faces (see Figure 4), while the cohesive interaction between the elements of the polymer chains decreases. This, apparently, leads to the fact that T_m_ decreases with an increase in the proportion of the filler in the unoriented samples.

**Figure 13:**
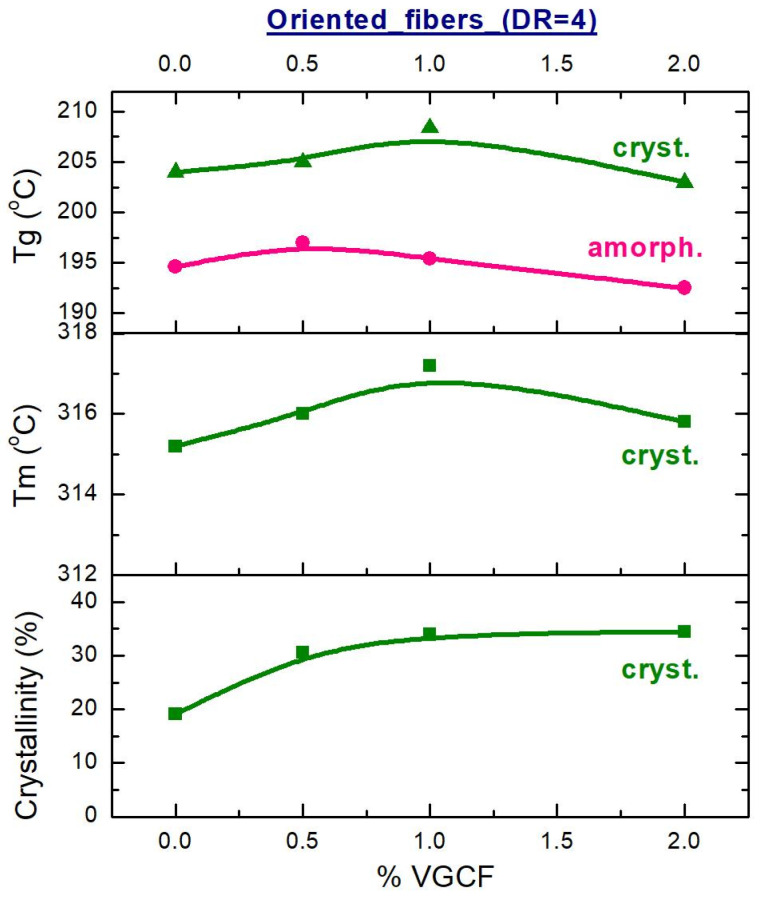
Dependences of the glass transition and melting temperatures, as well as the degree of crystallinity of the oriented composite fibers before (amorph.) and after crystallization (cryst.) on the content of the nanofiller.

The dependence of the size of the R-BAPB crystallites on the nanofillers concentration for the oriented (DR = 4) crystallized composite fibers, determined by the WAXS method (Figure 7), is similar in shape to the dependence T_m_ (%VGCF) shown in Figure 13 for the same samples, which indicates good agreement of the received data.

In the oriented state (DR = 4), the glass transition temperature (Tg) of the crystallized samples is higher than the Tg of the amorphous fibers by an average of 10 °C. Therefore, the mobility of the macromolecules in the crystallized samples is strongly inhibited. Nevertheless, the crystallite size first increases and then decreases depending on the content of the introduced nanofiller. But the degree of crystallinity remains unchanged. Therefore, it can be assumed that at a concentration of 1 wt.% VGCF, the polyimide matrix consists of large crystallites. With a further increase in the content of the nanoparticles, the crystallite size decreases, but the number of crystallites increases (since a larger number of the nucleating centers, such as nanoparticles, appears in the polymer), which ultimately gives the same volume fraction of the crystallized polymer.

### 3.5. Mechanical Properties

**Figure 14:**
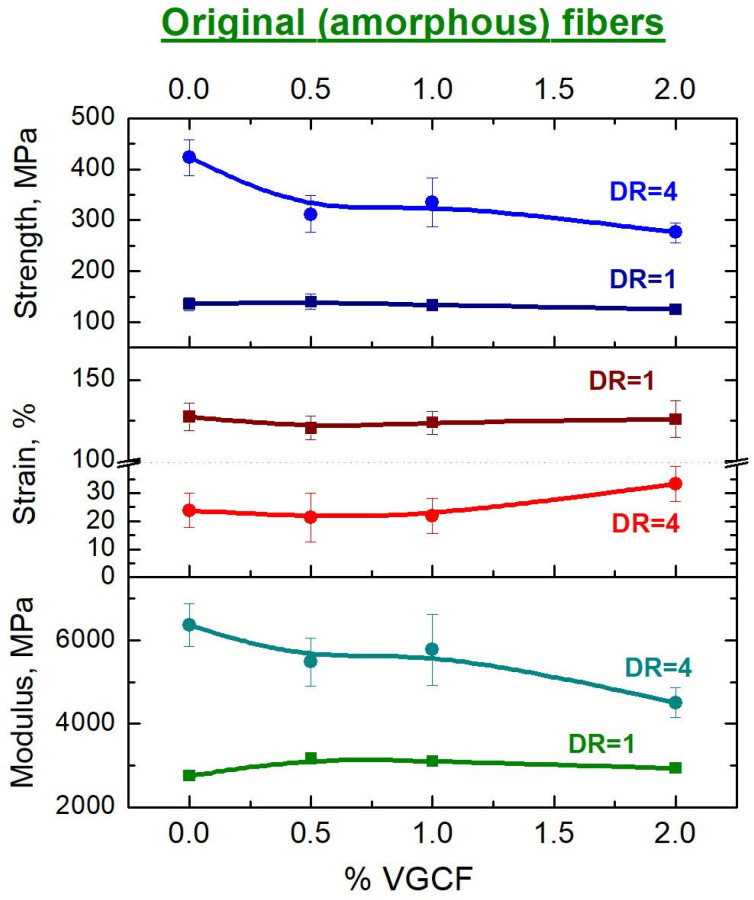
Dependences of the mechanical properties on the nanoparticle concentration in the polyimide matrix of the amorphous fibers before (DR = 1) and after (DR = 4) orientational drawing.

Figure 14 shows the concentration dependences of the mechanical characteristics (tensile strength, strain at break and Young’s modulus) of the amorphous composite fibers based on polyimide R-BAPB both before (DR = 1) and after orientational drawing (DR = 4).

It is clearly shown that orientational stretching results in a significant increase in the strength and elastic modulus of these composite fibers, comparing to the undrawn ones, but at the same time it also noticeably reduces the plasticity of the fibers (strain at break). It is also worth noting that at high concentrations of the nanoparticles (2 wt.% VGCF) introduced into the polyimide matrix, both the strength and the elastic modulus also decrease, which is especially noticeable for the oriented samples. This is probably due to the fact that the nanoparticles in such high concentrations being in the field of mechanical forces begin to interfere both with each other and the polymer macromolecules to be oriented along the fiber axis and, thereby, reduce the efficiency of orientational drawing. We would like also to remind that a similar effect has already been described earlier when considering the results of WAXS. In addition, it should be taken into account that melts of R-BAPB polyimide with a high content of the fillers had a high viscosity (see Figure 2) and, as a consequence, the composite fibers obtained in the process of extrusion could have a higher degree of jet drawing. Thus, the fibers were already overdrawn upon further high-temperature orientational drawing up to DR = 4 and, as a result, had an increased number of microdefects, chain breaks, etc. As mentioned above, according to SEM data, the nanoparticles loaded into the polyimide have low adhesion to the polymer matrix and, hence, can be considered as numerous defects. Consequently, the higher the nanoparticles content in the composite fiber, the higher the concentration of such defects. This, in our opinion, explains the decrease in strength and elastic modulus.

However, the strain at break on these fibers with the high content the nanoparticles tends to increase. We believe that the reason for this is also a significant defectiveness of such fibers and, therefore, an increase in plasticity is observed due to the facilitation of sliding of structural elements relative to each other during mechanical tests.

**Figure 15:**
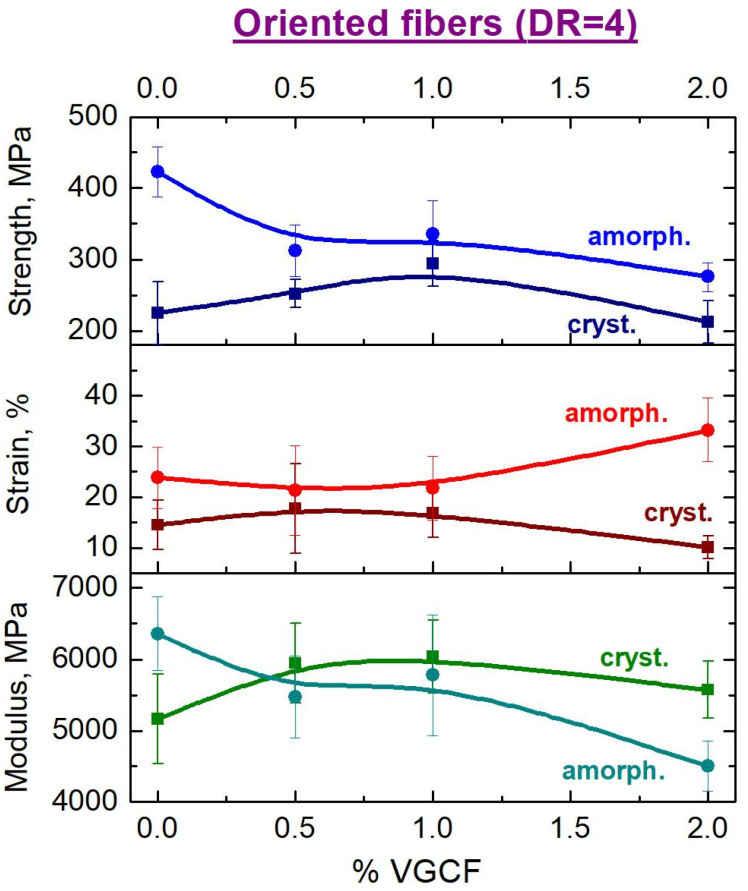
Dependences of the mechanical properties on the concentration of the VGCF nanoparticles in the R-BAPB polyimide matrix for the amorphous and crystallized fibers after orientational drawing (DR = 4).

Comparative dependences of the mechanical properties on the concentration of the nanoparticles in the oriented (DR = 4) composite fibers in both amorphous and crystallized states are shown in Figure 15. It can be well seen from the presented data that a change in the fine structure of the oriented fibers based on R-BAPB during crystallization leads, ultimately, to a decrease in almost all mechanical characteristics. We assume, that the formed crystallites are weakly connected to each other by through chains and, thus, the number of the load-bearing macromolecules is too small to withstand the applied stress. It can be supposed that after the formation of the crystallites, the disordered regions of the polymer matrix consist mainly of the ends of macromolecules located at the boundaries of the crystalline regions. Nevertheless, as it was expected, one can observe the maximum strength characteristics at the concentration of 1 wt.% VGCF in the polyimide matrix, since this fiber demonstrated the best alignment of the crystallites along the fiber axis (see Figure 7).

It should be noted that although the introduction of the carbon nanofibers does not lead to noticeable improvements in the mechanical properties compared to the pure amorphous R-BAPB fiber, the VGCF nanoparticles have a significant, very positive effect on the behavior of the fibers during crystallization; namely, the nanoparticles increase the crystallization rate and reduce the shrinkage of the oriented fibers during annealing, allowing to obtain the crystallized fiber without breaks.

It is worthy to note, that the mechanical characteristics (tensile strength and Young’s modulus) of the investigated R-BAPB based fibers exposed to orientation drawing are much higher than those described in the Introduction part [19,20,21,22].

Despite the obtained negative data on the study of the mechanical properties of the crystallized oriented composite fibers based on R-BAPB polyimide, it is worth noting the important fact that it is the crystallized fibers that have certain prospects in their use at high temperatures, since the level of their operation will be limited not by the glass transition temperature, as in the amorphous fibers, but by the melting temperature of the crystalline regions. Therefore, we believe that started and described above investigations of the R-BAPB polyimide fibers modified by the nanofiller should be continued to reveal mechanical behavior of these fibers at higher temperatures.

## 4. Conclusions

In the presented work, the R-BAPB polyimide-based fibers filled with the VGCF nanofibers were obtained by melt-extrusion method followed by orientational drawing up to DR = 4 and additional high-temperature thermal treatment. As a result, the fibers were prepared in amorphous and partially crystalline states. The composite polyimide fibers were investigated by a number of experimental techniques, i.e., SEM, WAXS, and DSC, as well as the mechanical properties were also examined.

It was clearly shown that the VGCF nanoparticles were able to change the internal structure of the nanocomposite fibers, since they can act as nucleating agents. It was also found that the introduced nanofiller can improve the orientation degree of the R-BAPB chains along the fiber axis. However, the high content of the nanoparticles (2 wt.% VGCF) could lead to increased misorientation of the crystalline part of the samples. The revealed changes in the fibers’ morphology affect the mechanical and thermal properties of the samples. Besides, the VGCF nanofibers were observed to improve the stability of the oriented samples being exposed to high temperature annealing.

## Data Availability

Not applicable.

## References

[1] Ding Y., Hou H., Zhao Y., Zhu Z., Fong H. (2016). Electrospun polyimide nanofibers and their applications. Prog. Polym. Sci..

[2] Mirjalili M., Karimi L. (2015). Preparation of melt spun electroconductive fine fibres containing carbon nanotubes. Autex Res. J..

[3] Bessonov M.I., Zubkov V.A. (1993). Polyamic Acids and Polyimides: Synthesis, Transformations, and Structure.

[4] Ha C.-S., Mathews A.S. (2011). Polyimides and High Performance Organic Polymers. Adv. Funct. Mater..

[5] Hicyilmaz A.S., Bedeloglu A.C. (2021). Applications of polyimide coatings: A review. SN Appl. Sci..

[6] Zhang M., Niu H., Wu D. (2018). Polyimide Fibers with High Strength and High Modulus: Preparation, Structures, Properties, and Applications. Macromol. Rapid Commun..

[7] Li W., Wu Z., Jiang H., Eashoo M., Harris F.W., Cheng S.Z.D. (1996). High-performance aromatic polyimide fibres. J. Mater. Sci..

[8] Vaganov G.V., Didenko A.L., Ivan’kova E.M., Popova E.N., Elokhovskii V.Y., Volkov A.V., Yudin V.E. (2020). Preparation and Properties of a Thermoplastic Partially Crystalline Polyimide in the Oriented State. Russ. J. Appl. Chem..

[9] Svetlichnyi V.M., Vaganov G.V., Myagkova L.A., Bugrov A.N., Chiryat’eva A.E., Vlasova E.N., Ivan’kova E.M., Elokhovskii Y.V., Popova E.N., Smirnova V.E. (2020). Electrospinning of Aqueous Solutions of a Triethylammonium Salt of Polyamic Acid and Properties of the Nonwoven Polyimide Materials. Russ. J. Appl. Chem..

[10] Yang S.-Y. (2018). Advanced Polyimide Materials: Synthesis, Characterization, and Applications.

[11] Xu Y., Wang S., Li Z., Xu Q., Zhang Q. (2013). Polyimide fibers prepared by dry-spinning process: Imidization degree and mechanical properties. Proc. J. Mater. Sci..

[12] Vaganov G., Didenko A., Ivan’kova E., Popova E., Elokhovskii V., Volkov A., Borisov I., Yudin V. (2020). Preparation of r-babp polyimide fibers via wet spinning of polyamide acid. Key Engineering Materials.

[13] Hufenus R., Yan Y., Dauner M., Kikutani T. (2020). Melt-spun fibers for textile applications. Materials.

[14] Verny L., Ylla N., Da Cruz-Boisson F., Espuche É., Mercier R., Sudre G., Bounor-Legaré V. (2020). Solvent-Free Reactive Extrusion As an Innovative and Efficient Process for the Synthesis of Polyimides. Ind. Eng. Chem. Res..

[15] Zheng S.S., Dong H., Wang S.H., Dong J., Guo T., Zhao X., Zhang Q.H. (2021). Scalable Reaction-spinning of Rigid-rod Upilex-S® Type Polyimide Fiber with an Ultrahigh Tg. Chin. J. Polym. Sci..

[16] Ivan’Kova E.M., Vaganov G.V., Popova E.N., Elokhovskiy V.Y., Kasatkin I.A. (2020). Structure-Property Relationship of Polyetherimide Fibers Filled with Carbon Nanoparticles. ACS Omega.

[17] Irwin R.S. (1983). Polyimide-Esters and Filaments. US Patent.

[18] Lienert K.-W. (1999). Poly (ester-imide) s for Industrial Use. Adv. Polym. Sci..

[19] Fay C.C., Hinkley J.A., St Clair T.L., Working D.C. (1998). Mechanical Properties of LaRC TM-IA and ULTEM R Melt-Extruded Fibers and Melt-Pressed Films. Adv. Perform. Mater..

[20] Vaganov G.V., Ivan’Kova E.M., Didenko A.L., Popova E.N., Elokhovsky V.Y., Yudin V.E. (2020). The study of heat-resistant fibers obtained from a melt of thermoplastic crystallizable polyimide. Proceedings of the Journal of Physics: Conference Series.

[21] Ivankova E.M., Vaganov G., Polyakov I.V., DIdenko A., Popova E.N. (2021). Influence of Carbon Nanoparticles on the Structure and Properties of Polyimide Crystallized Fibers Obtained by Melt-Spinning. Proceedings of the 2021 IEEE Conference of Russian Young Researchers in Electrical and Electronic Engineering, ElConRus.

[22] Yudin V.E., Svetlichnyi V.M. (2016). Carbon plastics based on thermoplastic polyimide binders modified with nanoparticles. Polym. Sci. Ser. C.

[23] Kumar S., Rath T., Mahaling R.N., Reddy C.S., Das C.K., Pandey K.N., Srivastava R.B., Yadaw S.B. (2007). Study on mechanical, morphological and electrical properties of carbon nanofiber/polyetherimide composites. Mater. Sci. Eng. Part B.

[24] Li B., Wood W., Baker L., Sui G., Leer C., Zhong W.H. (2010). Effectual dispersion of carbon nanofibers in polyetherimide composites and their mechanical and tribological properties. Polym. Eng. Sci..

[25] Hegde M., Lafont U., Norder B., Samulski E.T., Rubinstein M., Dingemans T.J. (2014). SWCNT induced crystallization in amorphous and semi-crystalline poly (etherimide) s: Morphology and thermo-mechanical properties. Polymer.

[26] Larin S.V., Nazarychev V.M., Dobrovskiy A.Y., Lyulin A.V., Lyulin S.V. (2018). Structural Ordering in SWCNT-Polyimide Nanocomposites and Its Influence on Their Mechanical Properties. Polymers.

[27] Larin S.V., Falkovich S.G., Nazarychev V.M., Gurtovenko A.A., Lyulin A.V., Lyulin S.V. (2014). Molecular-dynamics simulation of polyimide matrix pre-crystallization near the surface of a single-walled carbon nanotube. RSC Adv..

[28] Siochi E.J., Working D.C., Park C., Lillehei P.T., Rouse J.H., Topping C.C., Bhattacharyya A.R., Kumar S. (2004). Melt processing of SWCNT-polyimide nanocomposite fibers. Compos. Part B Eng..

[29] Yin C., Dong J., Li Z., Zhang Z., Zhang Q. (2014). Large-scale fabrication of polyimide fibers containing functionalized multiwalled carbon nanotubes via wet spinning. Compos. Part B Eng..

[30] Vaganov G., Didenko A., Ivan’kova E., Popova E., Yudin V., Elokhovskii V., Lasota I. (2019). Development of new polyimide powder for selective laser sintering. J. Mater. Res..

[31] Yudin V., Svetlichnyi V., Gubanova N., Didenko A., Popova E., Sukhanova T., Grigoriev A., Kostereva T., Arbel I., Marom G. (2005). Influence of crystallinity of R-BAPB-type polyimide matrix on thermal and mechanical properties of carbon-fiber-reinforced composites. Polyim. Other High Temp. Polym..

[32] Knauert S.T., Douglas J.F., Starr F.W. (2007). The effect of nanoparticle shape on polymer-nanocomposite rheology and tensile strength. J. Polym. Sci. Part B Polym. Phys..

[33] Huang Y.Y., Ahir S.V., Terentjev E.M. (2006). Dispersion rheology of carbon nanotubes in a polymer matrix. Phys. Rev. B -Condens. Matter Mater. Phys..

[34] Bassett D.C., Olley R.H. (1984). On the lamellar morphology of isotactic polypropylene spherulites. Polymer.

[35] Jiang X., Bin Y., Matsuo M. (2005). Electrical and mechanical properties of polyimide-carbon nanotubes composites fabricated by in situ polymerization. Polymer.

[36] Wunderlich B. (1980). Macromolecular Physics-Crystal Melting.

